# Groundwater quality and health risks for drinking and irrigation in densely populated Qalyubia, Egypt

**DOI:** 10.1038/s41598-026-58329-w

**Published:** 2026-06-28

**Authors:** Mohamed Elsayed Gabr, Hoda Soussa

**Affiliations:** 1 Civil Engineering Department, Higher Institute for Engineering and Technology, Ministry of Higher Education and Scientific research, New Damietta, Cairo Egypt; 2 Irrigation and Hydraulics Department, Faculty of Engineering, Ain Shams University, Cairo, Egypt

**Keywords:** Groundwater quality, Irrigation water indices, WQI, Potentially toxic elements, Metal index, Health hazard index, Egypt, Environmental sciences, Hydrology, Risk factors

## Abstract

This study comprehensively assesses groundwater suitability for drinking and irrigation and evaluates the associated non-carcinogenic health risks for children and adults in the urban, densely populated Qalyubia Governorate, South Nile Delta, Egypt. Groundwater samples from ten sites were analyzed for thirteen chemical parameters. An integrated approach to water quality assessment was applied, using the Water Quality Index (WQI), multiple irrigation indices, the Metal Index (MI), and the Heavy Metal Pollution Index (HPI) against World Health Organization (WHO) and Egyptian water quality standards. Key findings reveal an apparent yet explainable divergence between the indices: while the WQI (22–36) and irrigation indices indicated good overall water quality for both drinking and irrigation, the Metal Index (MI: 0.81–3.29) signaled a “moderately affected” status. This contrast underscores that the WQI, which integrates a broad suite of physicochemical parameters, can reflect generally good conditions, whereas the MI is specifically sensitive to the presence of even low concentrations of potentially toxic elements (PTEs) such as Fe, Mn, Cd, As, Cr, Pb, Hg, Ni, and Zn. The HPI values (≤ 15) indicated minimal pollution from PTEs. No significant non-carcinogenic health risks were identified for the analyzed metals, though children were more susceptible than adults. The study concludes that although the groundwater is generally suitable for use, the MI results provide a critical early warning of moderate metal pollution, necessitating regular monitoring and preemptive measures to safeguard public health and water resources.

## Introduction

Freshwater security represents one of the most pressing challenges of the 21st century, with groundwater resources providing nearly half of global drinking water and supporting 40% of irrigated agriculture. Their protection is fundamental to achieving multiple Sustainable Development Goals (SDGs), particularly SDG 6 (clean water and sanitation) and SDG 3 (good health and well-being)^1,2^. In arid and semi-arid regions, groundwater assumes even greater significance as climate change intensifies hydrological variability under SDG 13 (climate action)^3^. Egypt epitomizes this crisis, with renewable water resources plummeting far below the water poverty line while simultaneously confronting pollution from rapid urbanization, agricultural intensification, and industrial expansion. The Nile Delta aquifer, a critical buffer against scarcity, faces irreversible contamination without science-guided interventions^4^. Effective groundwater management requires integrated assessment frameworks that simultaneously evaluate hydrochemical suitability, contamination pathways, and human health risks^5,6^. This is particularly critical in rapidly urbanizing regions where anthropogenic pressures from industry, agriculture (fertilizers and pesticides), and untreated wastewater discharge introduce potentially toxic elements (PTEs) (e.g., Fe, Mn, Cr, As) and pathogens into aquifers^7,8^. A complete evaluation must consider the exposure pathway—the course a contaminant takes from the source to the point of human contact. This pathway includes the contaminant source, its fate and transport through the environment, the point of exposure (e.g., a household tap), the route of exposure (ingestion, dermal contact, or inhalation), and the potentially exposed population^9–11^. For groundwater, the primary exposure routes are ingestion of drinking water and dermal contact during bathing or swimming. To translate water quality data into actionable insights for risk assessment and management, a suite of complementary indices is essential, as no single metric can capture the multidimensional threats to groundwater^6^. This study employs an integrated framework of established techniques to provide a holistic diagnosis of the aquifer’s status: Water Quality Index (WQI): This index quantifies overall potability by aggregating key physicochemical parameters into a single value, providing an accessible measure of general water quality for the public and policymakers^12^. Irrigation indices (e.g., SAR, Magnesium hazard (MH) diagnose specific threats to agricultural productivity, such as salinity and sodicity, which can degrade soil structure and reduce crop yields^13^. Pollution Indices (MI and HPI): These track the cumulative impact of multiple heavy metals, which is crucial because contaminants can pose health risks even at low concentrations when mixed^14^. The Metal Index (MI) and Heavy Metal Pollution Index (HPI) provide a targeted assessment of toxic element pollution that might be obscured in a general WQI^15^. Health Risk Assessment: This evaluation quantifies the probability of adverse health effects by integrating contaminant concentration data with exposure analysis^16^. It considers factors such as the properties and toxicity of the contaminant, the duration and frequency of exposure, and the route of exposure (ingestion, dermal) to evaluate risks to different demographic groups, such as children versus adults. The Hazard Index (HI), Hazard Quotients (HQ), and the Hazardous Lifetime Cancer Risk (HLCR) translate contaminant concentrations into population-scale vulnerabilities, prioritizing mitigation^17,18^. The Nile Delta Aquifer, supplying about 10% of Egypt’s water (6.5 km³/year), faces severe strain from agricultural return flows, industrial effluents, and urban expansion^10,9^. The Qalyubia Governorate exemplifies the complex hydrogeological dynamics of the Nile Delta system. This densely populated region relies on shallow Quaternary aquifers recharged primarily through Nile seepage (≈ 60%), irrigation percolation (≈ 30%), and rainfall infiltration (≈ 10%)^4,20^. This recharge mechanism creates acute vulnerability: Pathogen infiltration from inadequate sewage systems elevates fecal coliform counts 100-fold beyond WHO limits in drainage-irrigated areas^4^. Heavy metal leaching from industrial discharges and agrochemicals introduces Fe, Mn, Cr, and Cd into groundwater pathways^20,21^. Salinization pressures from irrigation return flows increase sodium adsorption ratios (SAR) to 17.3, threatening soil structural stability^5,6^. Compounding these risks, aquifer overexploitation has drawn contamination plumes deeper into groundwater reserves, creating a “toxic legacy” for future generations^1^. In Qalyubia Governorate, groundwater dependency intensifies due to canal-end irrigation shortages^4^, yet contamination risks remain systematically unassessed. While WQI and irrigation indices have been applied locally^4,22^, integrated assessments linking hydrochemistry, pollution indices, and health risks are scarce for Egypt’s urbanizing delta critical gap given global evidence of metal-driven health crises in similar settings^23^. This study applies this integrated theory in Egypt’s Nile Delta, where groundwater security is critical for achieving UN Sustainable Development Goals. By applying this complementary suite of indices and a formal risk assessment framework, this research aims to provide a science-guided intervention to safeguard water resources, protect human health, and support sustainable development in the region. To address this, the groundwater suitability for drinking (via WQI) and irrigation (via SAR, MH, WSI) in Qalyubia is assessed. Quantify heavy metal pollution using HPI and MI. Model non-carcinogenic (HI/HQ) and carcinogenic (TR) health risks for adults/children. Establish a baseline for sustainable management within Integrated Water Resources Management (IWRM) frameworks. Methodological Alignment: Groundwater from 10 sites (2022) was analyzed for 13 parameters (Na⁺, K⁺, Ca²⁺, Mg²⁺, Fe, Mn, Cd, As, Cr, Pb, Hg, Ni, Zn) against the WHO^24^ and Egyptian drinking water quality standards^25^. Indices were computed to test the theory that multidimensional assessment reveals risks overlooked by conventional monitoring.

## Materials and methods

### Study area

**Fig. 1:**
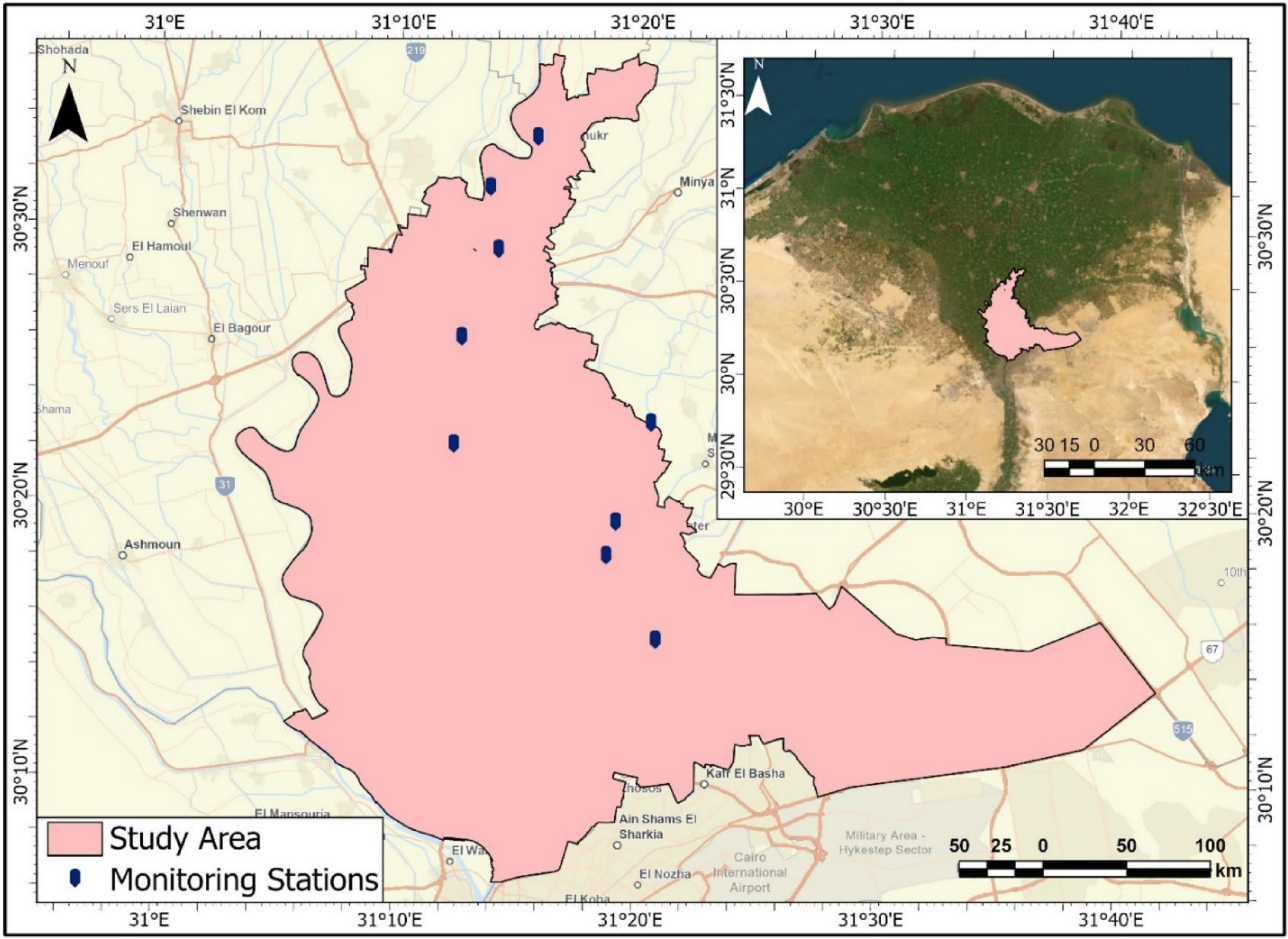
Location of the study area and groundwater sampling sites in Qalyubia Governorate, Egypt. The map was created by the authors using ArcGIS Pro 3.1 (ESRI, Redlands, CA, USA; https://www.esri.com/en-us/arcgis/products/arcgis-pro) with the projected coordinate system WGS 1984 UTM Zone 36 N.

**Fig. 2:**
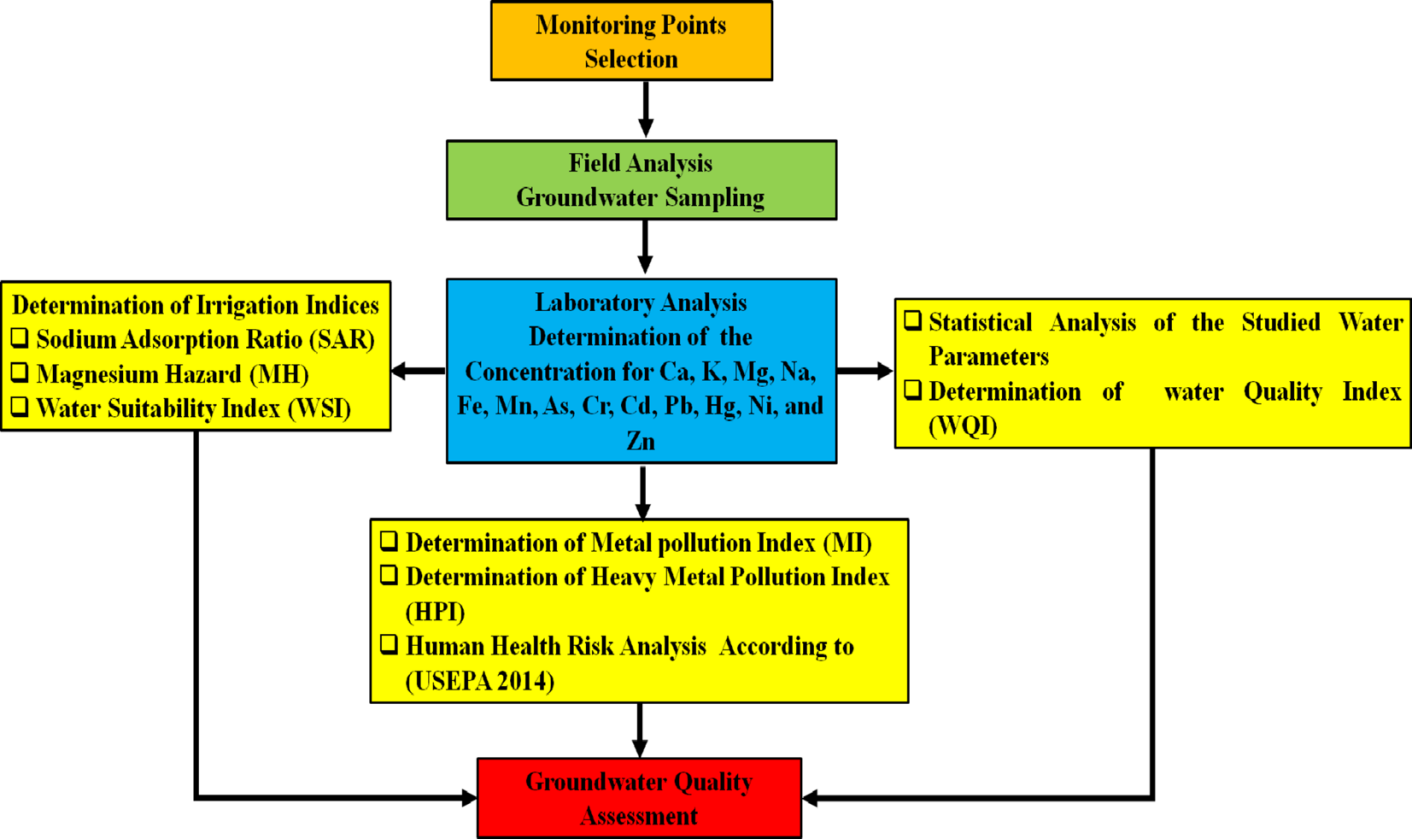
Research methodology.

The study was conducted in Qalyubia Governorate, Egypt, 29.93°N to 30.65°N and 31.07°E to 31.53°E, a densely populated agricultural and industrial hub in the eastern Nile Delta (Fig. 1). Ten groundwater wells, operated by the Egyptian Holding Company for Water and Wastewater (HCWW), were selected to represent diverse land-use zones, including proximity to industrial clusters (Al Obour, Al Safa, Al Shorouk, Al Aqrasha) and agricultural areas (Fig. 1). Sampling sites were strategically chosen to reflect contamination risks from urbanization, industrial effluents, and agricultural runoff. Table 1 summerizes the groundwater wells monitoring site coordinates. In addition, Fig. 2 shows the research methodology.

**Table 1:** Groundwater wells monitoring site coordinates.

SR.	Monitoring site	Latitude (*N*)	Longitude (E)
1	Site A	30^o^ 32^/^ 57^//^	31^o^ 15^/^ 42^//^
2	Site B	30^o^ 25^/^ 41^//^	31^o^ 12^/^ 38^//^
3	Site C	30^o^ 31^/^ 07^//^	31^o^ 13^/^ 45^//^
4	Site D	30^o^ 25^/^ 58^//^	31^o^ 17^/^ 15^//^
5	Site E	30^o^ 21^/^ 48^//^	31^o^ 12^/^ 22^//^
6	Site F	30^o^ 22^/^ 40^//^	31^o^ 20^/^ 35^//^
7	Site G	30^o^ 19^/^ 4^//^	31^o^ 19^/^ 10^//^
8	Site H	30^o^ 14^/^ 49^//^	31^o^ 20^/^ 53^//^
9	Site I	30^o^ 17^/^ 51^//^	31^o^ 18^/^ 47^//^
10	Site J	30^o^ 28^/^ 52^//^	31^o^ 14^/^ 7^//^

### Groundwater sampling and analysis

Triplicate groundwater samples were collected in December 2022 (winter) from a depth of 15 m following APHA^26^ protocols. A total of ten groundwater samples were collected from representative locations during the 2022 dry season. This sample size was determined based on a preliminary survey and a focused sampling strategy aimed at key zones with known or potential pollution sources (e.g., proximate to industrial drains, agricultural land receiving intensive fertilizer application, and densely populated urban centers). This targeted approach ensures that the samples capture the critical spatial variability of water quality parameters relevant to the study’s objectives. We acknowledge that this limits extensive geostatistical analysis, but it provides a robust preliminary assessment of contamination hotspots, which is valuable for guiding future, more extensive monitoring campaigns. In addition, the present study focused on major cations and trace potentially toxic elements (PTEs) to specifically assess the impact of anthropogenic activities. Several previous investigations conducted in the same study area or in comparable hydrogeological settings have demonstrated that the major anions generally fall within permissible limits and that groundwater quality is largely suitable for irrigation and domestic uses. For example, the comprehensive study by Eltarabily et al.^17^ reported that groundwater in the area is predominantly alkaline, characterized mainly by bicarbonate and sodium water types, with total dissolved solids (TDS) values below 2500 mg/L. Their results showed acceptable salinity levels, low sodium hazard (SAR < 10), low residual sodium carbonate (RSC < 1.5), and overall suitability for irrigation purposes. Furthermore, multivariate statistical analysis in that study indicated that hydrochemical variability was primarily controlled by salinization and agricultural activities rather than anion-related contamination. Based on these well-established findings, and to avoid redundancy, the present study did not re-measure major anion concentrations. Instead, it focuses on heavy metals, which represent the most critical and persistent contaminants of concern due to their toxicity, non-biodegradability, and potential long-term impacts on human health and the environment. Wells were purged to remove stagnant water, ensuring representative aquifer samples. Samples were stored in acid-washed polyethylene bottles and analyzed at Ain Shams University’s water quality laboratory for:

1. Major ions (Ca²⁺, Mg²⁺, Na⁺, K⁺): Measured via flame photometry (Jenway PHF 80B) and atomic absorption spectrometry (Perkin-Elmer 2380).
2. PTEs (Fe, Mn, Cd, As, Cr, Pb, Hg, Ni, Zn): Quantified using ICP-MS (Thermo Jarrell POEMSIII) and atomic absorption spectrometry (APHA Method 3111).
3. Alkalinity: Assessed via titration with methyl orange and phenolphthalein indicators.
4. Quality assurance included GF/C filtration, calibration with 1000 mg/L standards, and adherence to a ± 5% ion balance error threshold.

### Irrigation and drinking water quality indices

#### Water quality index (WQI)

The WQI is a numerical indicator used to assess water quality for various purposes^12^. The WQI was computed using Egyptian drinking water standards^25^ and the WHO^24^ standards (Table 2). Relative weights (*Wi*) and sub-indices (*SIi*) were derived as:

1 $$\:WQI=\sum\:_{1}^{n}\left(\frac{wi}{\sum\:wi}\times\:\frac{Ci}{Si}\times\:100\right)\:$$

where *wi*: Weight assigned to the *i*-th parameter (Table 2), *Ci*: Measured concentration of the *i*-th parameter (mg/L), *Si*: Regulatory standard^24,25^ for the *i*-th parameter (mg/L), and *n*: Total number of parameters analyzed (13 in this study). According to the WQI, classification followed^13^: 0–25: Excellent, 26–50: Good, 51–75: Poor, 76–100: Very poor, and > 100: Unsuitable. To comprehensively assess the suitability of groundwater for irrigation, key indices including the Sodium Adsorption Ratio (SAR), Soluble Sodium Percentage (SSP), Kelly’s Ratio (KR), Magnesium Hazard (MH), Permeability Index (PI), and Total Hardness (TH) will be determined. These indices collectively evaluate critical water quality aspects such as sodium hazard (risk of soil sodicity and permeability reduction), magnesium dominance (potential soil imbalance), and overall mineral content affecting soil structure and plant health. The calculated values for each index will be interpreted according to established classification schemes to determine irrigation suitability, with the specific threshold ranges and categories for each index^13,14,27,28^ summarized in Table 3.

**Table 2:** Groundwater quality weight based on WHO^24^ and the Egyptian water quality guidelines^25^.

Metals	Symbol	Unit	WHO^24^	EWQG^25^	Assigned weight	Relative weight
Calcium	Ca	mg/L	75	75	2	0.033
Potassium	K	mg/L	12	12	2	0.033
Magnesium	Mg	mg/L	50	30	2	0.033
Sodium	Na	mg/L	200	200	3	0.050
Iron	Fe	mg/L	0.3	0.3	4	0.067
Manganese	Mn	mg/L	0.4	0.4	5	0.083
Arsenic	As	mg/L	0.01	0.01	5	0.083
Cadmium	Cd	mg/L	0.003	0.003	5	0.083
Chromium	Cr	mg/L	0.05	0.05	5	0.083
Lead	Pb	mg/L	0.01	0.01	5	0.083
Mercury	Hg	mg/L	0.001	0.001	5	0.083
Nickel	Ni	mg/L	0.07	0.02	5	0.083
Zinc	Zn	mg/L	3	3	3	0.050
Bicarbonate	HCO_3_	mg/L	500	500	2	0.033
Chloride	Cl	mg/L	600	600	2	0.033
Sulfate	SO_4_	mg/L	600	600	2	0.033
Nitrate	NO_3_	mg/L	50	50	3	0.050
				sum	59	1

**Table 3:** Classification of the irrigation indices for the groundwater uses in the irrigation purposes.

Parameters	Class	Range	Classification	References
Sodium Adsorption Ratio (SAR)	C1 C2 C3 C4	$\:<$ 10 mg/L 10–18 mg/L 18–26 mg/L $\:>\:$26 mg/L	Excellent Good Doubtful Unsuitable	Richards^13^
Soluble sodium percentage (SSP)	C1 C2 C3 C4 C5	< 20% 20–40% 40–60% 60–80% > 80%	Excellent Good Permissible Doubtful Unsuitable	Todd^27^
Kelly’s Ratio (KI)	C1 C2	$\:\:\:\:\:\:\:\:\:\:\:\:\:<1$ $\:>$ 1	Suitable Excess level	Kelley^28^
Magnesium hazard (MH)	C1 C2	$\:<50$ mg/L $\:>\:$50 mg/L	Excellent Harmful for soil	Gautam et al.^14^
Total Hardness (TH)	C1 C2 C3 C4	0–60 meq/L 61–120 meq /L 121–180 meq /L $\:>$180 meq /L	Soft Moderately hard Hard Very hard	Todd^27^

### Sodium adsorption ratio (SAR)

According to Gautam et al.^14^, SAR determines the appropriateness of groundwater for irrigation. SAR is the ratio of Na^+^, Ca^2+^, and Mg^2+^ levels in the water, indicating the rate of sodium absorption by the soil. According toWilcox^29^ the SAR is given by Eq. (2).

2 $$\:SAR=\:\frac{{Na}^{+}}{\sqrt{({Ca}^{2+}+{Mg}^{2+})/2}}$$

### Magnesium hazard (MH)

Magnesium hazard value is crucial for irrigation uses^29^. Equation (6) describes how it is calculated.

3 $$\:MH=\:\frac{{Mg}^{2+}}{{Ca}^{2+}+{Mg}^{2+}}\:\times\:100$$

### Metal index (MI) and heavy metal pollution index (HPI)

The MI is based on a comprehensive trend analysis of the current grade. Higher metal concentrations compared to their respective maximum allowable concentration (MAC) values lead to poorer water quality. According to^30^, a metal index (MI) scores greater than 1 indicates a warning. MI was calculated as follows:

4 $$\:MI=\:\sum\:_{i=1}^{n}\frac{{C}_{i}}{MA{C}_{i}}$$

Where $\:{C}_{i}$ is the level of each metal and $\:MA{C}_{i}$ maximum allowable concentration of each metal. Table 4 summarizes water quality classification according to the metal index (MI).

**Table 4:** Water quality classification according to the metal index (MI).

Class	Property	Metal index MI values
I	Very Pure	$\:<0.3$
II	Pure	0.3-1
III	Slightly Affected	1–2
IV	Moderately Affected	2–4
V	Strongly Affected	6
VI	Seriously Affected	$\:>6$

The Heavy Metal Pollution Index (HPI) is a number to evaluate the overall influence of several heavy metal indicators on water quality. Scientists use the HPI metric to quantify the total impact of PTEs pollution on water quality^11^. HPI is determined according to^31^ as follows:

5 $$\:HPI=\:\frac{{\sum\:}_{i=1}^{n}\left({Q}_{i}{W}_{i}\right)}{{\sum\:}_{i=1}^{n}{{S}_{i}W}_{i}}$$

6 $$\:{W}_{i}=\:\frac{K}{{S}_{i}}$$

7 $$\:{Q}_{i}=\:\frac{{M}_{i}}{{S}_{i}}$$

Where Wi is the unit weight of metals, Qi represents the sub-index of each metal, Mi represents the monitored value of PTEs, WHO^24^ approved standard values for Si parameters in drinking water, and “k” represents a constant equal to one. The HPI values are characterized into 3 groups: (i) the HPI score is less than 15, the pollution level is considered low, (ii) the HPI value between 15 and 30 shows a moderate level of pollution, and (iii) a high HPI value > 30.

### Water suitability index (WSI)

The water suitability index is a mixture of WQI, SAR, and MH^14^, indicating a simple average of the three indices. The WSI value and appropriateness for irrigation and drinking are inversely related. As a result, the lower the value, the better the suitability, and the higher the value, the less suitable for irrigation and drinking. Equation (8) shows how it’s calculated.

8 $$\:WSI=\:\sum\:(WQI+SAR+MH)/3$$

### Total hardness (TH)

The total hardness in meq/L is given by^27^:

9 $$\:TH=2.497\:{Ca}^{2+}+4.11\:{Mg}^{2+}$$

### Soluble sodium percentage (SSP)

The soluble sodium percentage in mg/L is given by^27^:

10 $$\:\mathrm{N}\mathrm{a}\mathrm{\%}=\:\frac{{Na}^{+}+{\:K}^{2+}}{{Ca}^{2+}+\:{Mg}^{2+}+{\:Na}^{+}+\:{K}^{2+}}\:\times\:100$$

### Kelly’s index (KI)

The Kelly’s index is given by Kelley^28^:

11 $$\:KI=\:\frac{{Na}^{+}}{{Ca}^{2+}+{\:Mg}^{2+}}$$

### Health risk overview

Through contact with freshwater and digestion (by drinking water), PTEs enter the human body through the epidermis. The potential health impacts of ingesting and cutaneous exposure, including both non-carcinogenic and carcinogenic implications, can be examined using experimental models. The health risk assessment method developed by the USEPA^32^ was used in the current investigation. The toxicological consequences of specific metals are listed in Table 5. For chronic daily intake via direct consumption (CDI _ingestion_) and skin absorption (CDI _dermal_) were calculated using formulas 12 and 13, respectively^33^.

12 $$\:{CDI}_{ingestion}=\:\frac{{C}_{water}\times\:IR\times\:EF\times\:ED\:\:\:\:}{BW\times\:AT}$$

13 $$\:{CDI}_{dermal}=\:\frac{{ET\times\:SA\:\times\:\:PC\times\:\:C}_{water}\times\:\:EF\times\:ED\:\times\:CF\:\:\:}{BW\times\:AT}$$

Where $\:{CDI}_{ingestion}$ is the chronic daily intake via ingestion (mg/kg/day), $\:{CDI}_{dermal}$ is the chronic daily intake via dermal (mg/kg/day), $\:{C}_{water}$ is the concentration of the contaminant in water (mg/L), IR is the ingestion rate (L/day) taken in this study for adults and children 2 L/day and 1 L/day respectively, SA is the skin surface area available for contact (cm²) taken in this study for adults and children 18,000 cm^2^ and 7000 cm^2^ respectively, PC is the dermal permeability constant (cm/hour) equal to 0.01 in this study, ET is the exposure time (hours/day) for dermal contact taken in this study for adults and children 0.5 h/day and 0.7 h/day respectively, $\:EF$ is exposure frequency (days/year) for both routes taken 350 days, $\:ED$ is the exposure duration (years) for both routes, $\:AT$ is the average time (days) for the both routes, in this study for carcinogenic assessments AT for adults is typically 70 years $\:\times\:$365 days/year, and for non-carcinogenic it is ED $\:\times\:$ 365 days/year whereas for children AT is typically 10 years $\:\times\:$365 days/year, and for non-carcinogenic it is ED $\:\times\:$ 365 days/year, $\:BW$ is the body weight (kg) taken in this study for adults and children 70 kg and 30 kg respectively, and CF is the conversion factor equal to 0.001. To quantify the potential noncarcinogenic effects of PTEs absorbed or penetrated the epidermis, the hazard quotient (HQ) and hazard index (HI) were calculated using Eqs. (14) and (15), respectively.

14 $$\:HQ={HQ}_{ingestion}+\:{HQ}_{dermal}=\:\frac{{ADD}_{ingestion}}{{RFD}_{ingestion}}+\:\:\frac{{ADD}_{dermal}}{{RFD}_{dermal}}$$

15 $$\:HI=\sum\:_{i=1}^{n}HQ={HQ}_{Fe}+{HQ}_{Mn}+{HQ}_{Cd}+{HQ}_{Cr}+{HQ}_{Pb}+{HQ}_{Ni}+{HQ}_{Se}+\:{HQ}_{Zn}+{HQ}_{Hg}$$

The hazard index (HI) was used to assess the non-carcinogenic risk to human health from exposure to multiple potentially toxic trace elements. The HI is the sum of all HQ calculated for individual trace elements. However, for HQ less than one indicates that the risk of negative health outcomes is low. On the other hand, the HI greater than one indicates that heavy metal exposure may not cause cancer. Hazardous Lifetime Cancer Risk (HLCR) evaluates a person’s lifetime risk of developing cancer as a result of exposure to carcinogenic substances. This risk is determined as:

16 $$\:HLCR=ADD\times\:CSF$$

CSF is often referred to as the cancer slope factor. In this study, cancer risk was calculated based on arsenic levels. Khalili et al.^33^ discovered that CSF levels are 0.0015 and 0.00366 mg/kg/day for ingestion and cutaneous penetration, respectively. Table 5 summarizes the toxicological parameters of the metals studied for health risk assessment, including reference dose (RFD) and cancer slope factor.

**Table 5:** Toxicological parameters of the metals under investigation were used to assess health risk, including reference dose (RfD) and cancer slope factor (CSF).

Element	PC	$\:{\mathrm{R}\mathrm{f}\mathrm{D}}_{\mathrm{i}\mathrm{n}\mathrm{g}\mathrm{e}\mathrm{s}\mathrm{t}\mathrm{i}\mathrm{o}\mathrm{n}}$ (m$\:\mathrm{g}/\mathrm{k}\mathrm{g}/\mathrm{d}\mathrm{a}\mathrm{y})$	$\:{\mathrm{R}\mathrm{f}\mathrm{D}}_{\mathrm{d}\mathrm{r}\mathrm{e}\mathrm{m}\mathrm{a}\mathrm{l}}$ (m$\:\mathrm{g}/\mathrm{k}\mathrm{g}/\mathrm{d}\mathrm{a}\mathrm{y})$	CSF (mg/kg/day)
Fe	0.01	700	140	–
Mn	0.01	24	0.96	–
As	0.01	0.3	0.285	
Cd	0.01	0.5	0.025	6.1
Cr	0.01	3	0.075	41
Pb	0.01	1.4	0.42	8.5
Hg	0.01	0.3	0.021	
Ni	0.01	20	0.8	0.84
Zn	0.01	300	60	

PC, the permeability coefficient in water; RfD _ingestion_, ingestion reference dose; RfD _deremal_, dermal reference dose; and CSF, cancer slope factor.

Equation (17) can be used to calculate the cumulative cancer risk caused by exposure to multiple carcinogenic, potentially toxic trace elements through water consumption. In this study, the cumulative cancer risk for Cd, Cr, Pb, and Ni was considered.

17 $$\:HLCR=\sum\:_{i=1}^{4}HLCR={HLCR}_{Cd}+{HLCR}_{Cr}+{HLCR}_{Pb}+{HLCR}_{Ni}$$

According to^34^ the acceptable cancer risk (*HLCR*) for regulatory purposes ranges from.

10^− 6^ to10^− 4^.

## Results

### Chemical parameters

Table 6 summarizes the statistical analysis of 13 chemical parameters (Ca, K, Mg, Na, Fe, Mn, As, Cr, Cd, Pb, Hg, Ni, Zn) in groundwater samples. All values were compared against drinking water standards^24,25^ to assess suitability.

### Cations (Na^+^, K^+^, Mg^2+^, and Ca^2+^) levels

**Fig. 3:**
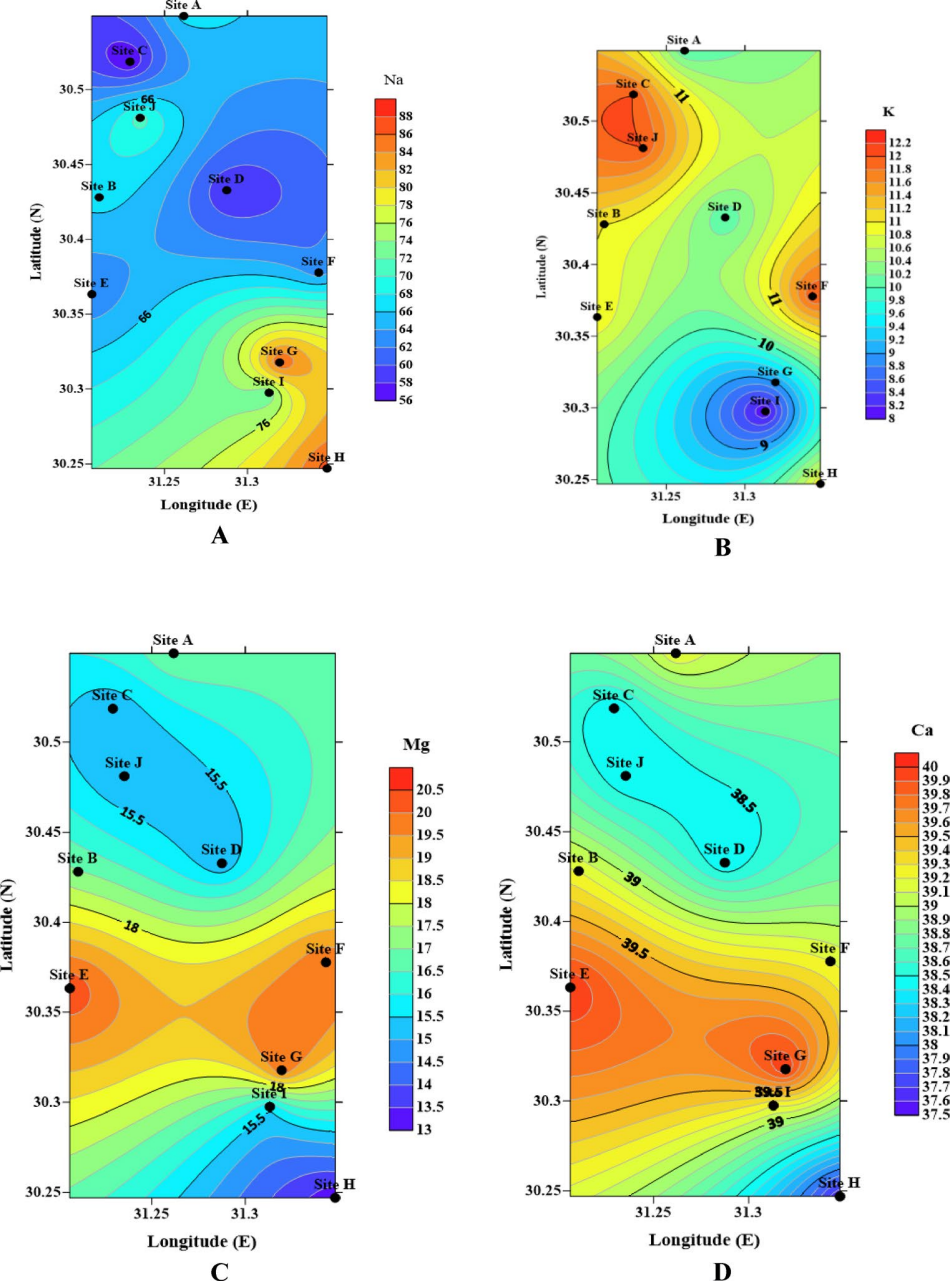
Cations concentration distribution in the study area (**A**): Na^+^, (**B**): K^+^, (**C**): Mg^2+^, and (**D**): Ca^2+^.

Na⁺ shows an average concentration of 69 ± 10.15 mg/L (range: 55.8–87 mg/L). All sites were below the 200 mg/L threshold (WHO/EWQG). Sites G and H showed the highest levels (Fig. 3A). K⁺: Average: 10.6 ± 1.28 mg/L (range: 8–12 mg/L). Sites C, J, and F exceeded 12 mg/L, nearing the WHO danger threshold (Fig. 3B). Mg²⁺: Average: 16.8 ± 2.38 mg/L (range: 13.14–20.44 mg/L). All sites met standards (Fig. 3C). Ca²⁺: Average: 38.96 ± 0.72 mg/L (range: 37.6–40 mg/L). All samples complied with the 75 mg/L limit (WHO/EWQG) (Fig. 3D). These cation results align with^17^.

**Table 6:** Statistics of the chemical components of groundwater samples.

Parameter	Unit	Site A	Site B	Site C	Site D	Site E	Site F	Site G	Site H	Site I	Site J	Min	Max.	Ave.	S.D.
Ca	mg/L	39.2	39.2	38.4	38.4	40	39.2	40	37.6	39.2	38.4	37.6	40	38.96	0.72
K	mg/L	10	11	12	10	11	12	9	11	8	12	8	12	10.6	1.28062
Mg	mg/L	17.03	17.03	15.09	15.09	20.44	19.95	19.95	13.14	15.57	15.09	13.14	20.44	16.838	2.38468
Na	mg/L	66.8	67	55.8	58	62.4	63	86.5	87	72.5	71	55.8	87	69	10.1496
Fe	mg/L	0.36	0.33	0.26	0.24	0.33	0.32	0.38	0.012	0.19	0.16	0.012	0.38	0.2582	0.10704
Mn	mg/L	0.45	0.42	0.31	0.29	0.45	0.46	0.51	0.01	0.26	0.14	0.01	0.51	0.33	0.15218
As	mg/L	0.0001	0.0001	0.0001	0.0001	0.0001	0.0001	0.0001	0.0001	0.0001	0.0001	0.0001	0.0001	0.0001	-
Cd	mg/L	0.001	0.001	0.001	0.001	0.001	0.001	0.003	0.003	0.001	0.001	0.001	0.003	0.0014	0.0008
Cr	mg/L	0.001	0.001	0.001	0.001	0.001	0.001	0.001	0.001	0.001	0.001	0.001	0.001	0.001	-
Pb	mg/L	0.0001	0.0001	0.0001	0.0001	0.0001	0.0001	0.0001	0.0001	0.0001	0.0001	0.0001	0.0001	0.0001	-
Hg	mg/L	0.015	0.015	0.019	0.019	0.018	0.018	0.019	0.019	0.018	0.018	0.015	0.019	0.0178	0.00147
Ni	mg/L	0.56	0.56	0.48	0.48	0.52	0.52	0.52	0.52	0.46	0.46	0.46	0.56	0.508	0.03487
Zn	mg/L	10	11	12	10	11	12	9	11	8	12	8	12	10.6	1.28062
HCO_3_	mg/L	300	350	340	300	280	300	290	350	320	300	280	350	313	24.1039
Cl	mg/L	38	38	32	32	62	58	76	28	38	34	28	76	43.6	15.1473
SO_4_	mg/L	69.2	68.6	58.2	61.2	84.2	79.2	89.2	51.2	62.3	55.4	51.2	89.2	67.87	12.0612
NO_3_	mg/L	2.8	2.9	1.1	1.06	2.6	2.5	3.2	1.16	1.3	1.28	1.06	3.2	1.99	0.83106

### Heavy metal levels

**Fig. 4:**
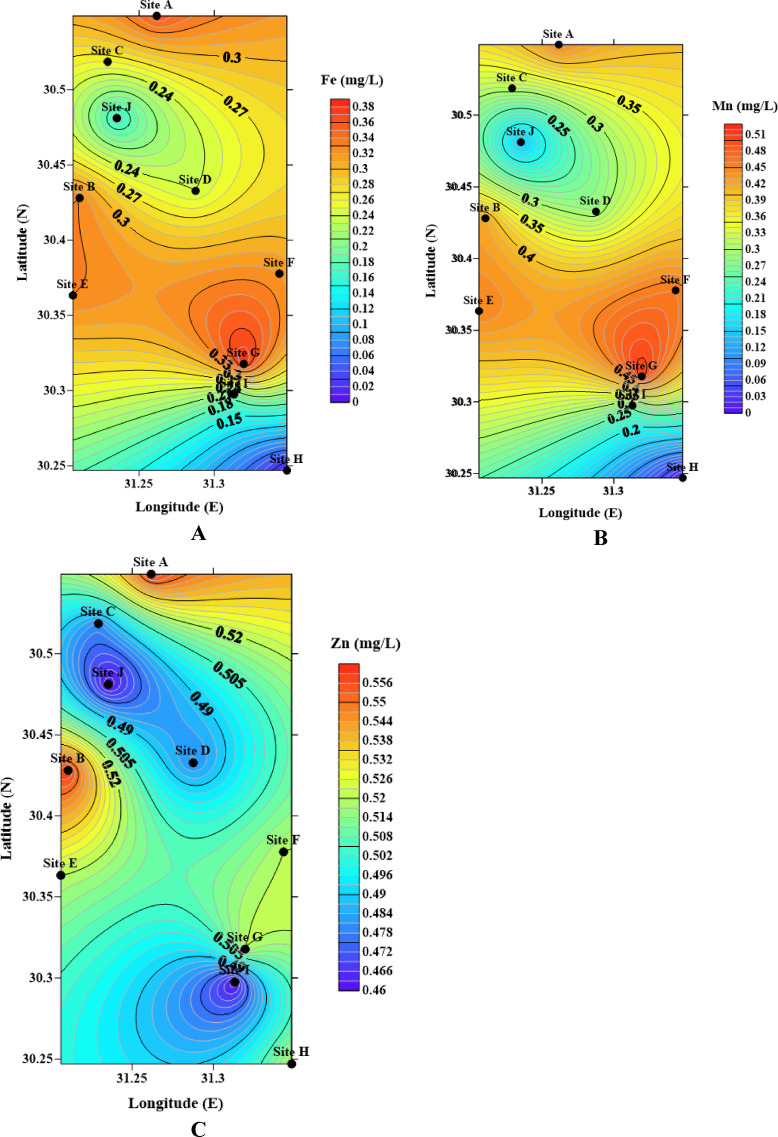
Heavy meatal concertation distribution in the study area, (**A**): Fe, (**B**): Mn, and (**C**): Zn.

Fe indicated an average of 0.26 ± 0.26 mg/L (range: 0.012–0.38 mg/L). Sites A, B, F, G, and H slightly exceeded limits (Fig. 4A). Anthropogenic sources (mining, agriculture, industrial waste) drive Fe contamination^6,15^. Excess Fe links to liver cirrhosis, hemochromatosis, and lipid damage^35,36^. Other Metals: Ni (average: 0.0178 ± 0.00147 mg/L), Cr (average: 0.0014 ± 0.0008 mg/L), Pb (average: 0.001 mg/L), As (average: 0.0001 mg/L), Hg (average: 0.0001 mg/L) all complied with standards. Sources include industrial emissions, mining, and vehicle exhaust^21^,36. Chronic exposure to Cr/Ni may cause cancer, kidney disease, or respiratory damage^36^. Mn: Average: 0.33 ± 0.15 mg/L (range: 0.01–0.51 mg/L). Sites A, B, F, G, and H exceeded the 0.4 mg/L limit (Fig. 4B). Overexposure causes skeletal defects, neurological issues, and reproductive harm^15^. Zn: Average: 0.51 ± 0.03 mg/L (range: 0.46–0.56 mg/L). Sites A, B, F, G, H, and I surpassed thresholds (Fig. 4C). Excess Zn induces vomiting, diarrhea, and neurological damage^15^. Elevated Fe, Mn, and Zn are attributed to agricultural activities. Fe trends match^17^.

### The water quality index (WQI)

**Fig. 5:**
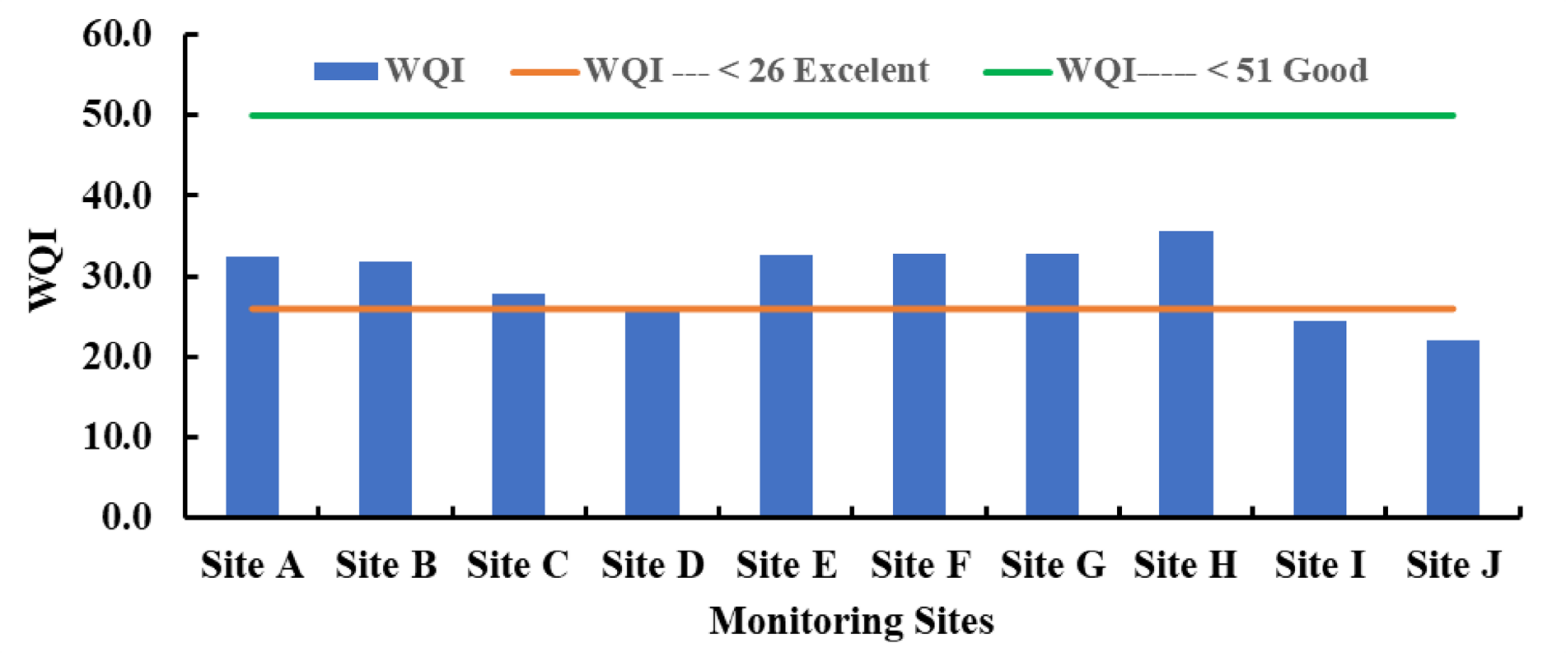
WQI for the study area.

WQI values ranged from 22 to 36 (Fig. 5): Sites I and J: Grade A (excellent; suitable for all uses). Other sites: Grade B (good; suitable for domestic/industrial/irrigation).

### Irrigation water indices

Table 7 summarizes key indices: SAR: Avg: 13.1 ± 2 mg/L (range: 10.8–17.3 mg/L). All sites indicate low sodium hazard (good irrigation suitability). MH: Avg: 30 ± 2.6 mg/L (range: 29.9–33.8 mg/L). Classified as Class C1 (excellent). Highest at Sites E, F, H (Table 7). WSI: Avg: 28 ± 2.5 (range: 24–32). All sites are suitable for irrigation/drinking. KI: Avg: 1.24 ± 0.21 (range: 1.03–1.71). Values > 1 suggest marginal suitability. TH: Avg: 102.98 ± 2.55 meq/L (range: 98.33–106.79 meq/L). Indicates moderately hard water. SSP: Avg: 0.59 ± 0.03 mg/L (range: 0.55–0.66 mg/L). Confirms good irrigation quality.

**Table 7:** Irrigation indices result for the studied groundwater samples.

Indicators	Unit	Site A	Site B	Site C	Site D	Site E	Site F	Site G	Site H	Site I	Site J	Min.	Max.	Ave.	S.D.
SAR	(mg/L)	12.6	12.6	10.8	11.2	11.4	11.6	15.8	17.3	13.9	13.7	10.8	17.3	13.1	2
MH	(%)	30.3	30.3	28.2	28.2	33.8	33.7	33.3	25.9	28.4	28.2	25.9	33.8	30	2.6
WSI	-	25.9	25.5	22.8	22.4	26.7	26.8	28.1	27.1	22.5	21.6	19.8	29.8	24.9	3.1
KI	%	1.19	1.19	1.04	1.08	1.03	1.07	1.44	1.71	1.32	1.33	1.03	1.71	1.24	0.21
TH	meq/L	103.64	103.64	100.99	100.99	106.79	104.63	106.63	98.33	103.15	100.99	98.33	106.79	102.98	2.55
SSP	mg/L	0.58	0.58	0.56	0.56	0.55	0.56	0.61	0.66	0.60	0.61	0.55	0.66	0.59	0.03

SAR, sodium adsorption ratio; SSP, soluble sodium percentage; KI, Kelly’s Ratio; NH, Magnesium hazard; WSI, water suitability index; and TH, total hardness.

### Metal index (MI) and heavy metal pollution index (HPI)

Metal Index (MI): Site H: 0.81 (Grade II: pure). Sites I/J: 1.56–1.96 (Grade III: slightly affected).

**Fig. 6:**
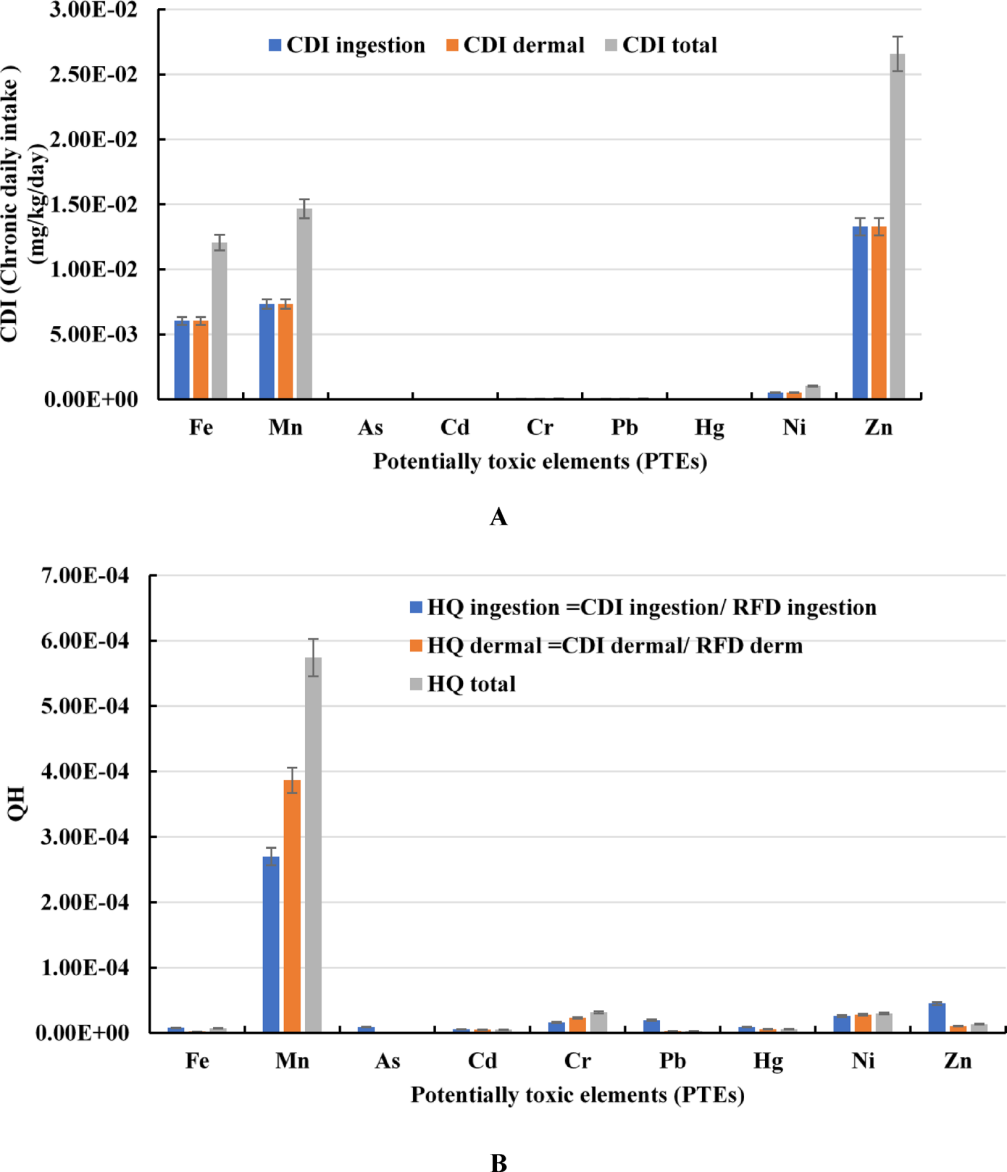
(**A**): Chronic daily intake (CDI) and (**B**): hazard quotient (HQ) via ingestion and dermal for adults.

Sites A–G: >1.96 (Grade IV: moderately affected) (Table 8). Heavy Metal Pollution Index (HPI): All metals showed HPI ≤ 45, indicating low pollution (Fig. 6).

**Table 8:** Metal Index (MI) results for the 10 groundwater sites.

Station	Metal Index = Ci/MAC	Class	Properties
Site A	2.99	IV	Moderately affect
Site B	2.81	IV	Moderately affect
Site C	2.34	IV	Moderately affect
Site D	2.22	IV	Moderately affect
Site E	2.92	IV	Moderately affect
Site F	2.91	IV	Moderately affect
Site G	3.29	IV	Moderately affect
Site H	0.81	IV	Pure
Site I	1.96	III	Slightly affect
Site J	1.56	III	Slightly affect

### Hazard quotient (HQ) and hazard index (HI)

Figure 6 shows the chronic daily intake (CDI) and the hazard quotient (HQ) via ingestion and dermal exposure for adults. In addition, Fig. 8 shows the chronic daily intake (CDI) and the hazard quotient (HQ) via ingestion and dermal exposure for children.

### Non-carcinogenic risks (HQ/HI)

For adults, the chronic daily intake (CDI): Ingestion: Highest for Mn (0.014 mg/kg/day) and the lowest for Cd and Hg (2.74$\:\times\:$10^−6^ mg/kg/day). Metal contribution to CDI ingestion: Zn > Mn > Fe > Ni > Cr > Pb > As > Cd > Hg. Dermal: Highest for Zn (1.53 $\:\times\:$ 10⁻^2^ mg/kg/day), lowest for Hg (2.74$\:\times\:$10^−6^ mg/kg/day). Metal contribution to CDI dermal: Zn > Mn > Fe > Ni > Cr > Pb > As > Cd > Hg. On the other hand, for children, the chronic daily intake (CDI): Ingestion: Highest for Zn (0.018 mg/kg/day) and the lowest for Cd and As (3.2$\:\times\:$10^−6^ mg/kg/day). Metal contribution to CDI ingestion: Zn > Mn > Fe > Ni > Cr > Pb > As > Cd > Hg. Dermal: Highest for Zn (8.77 $\:\times\:$ 10⁻⁴ mg/kg/day), lowest for Hg (1.57$\:\times\:$10^−7^ mg/kg/day). Metal contribution to CDI dermal: Zn > Mn > Fe > Ni > Cr > Pb > As > Cd > Hg.

### Hazard quotient (HQ) & hazard index (HI)

For adults, all metals showed HQ < 1 and the Hazard Quotient for the ingestion and dermal (HI_total_) indicated a value of 9.22$\:\times\:$10^−4^ (≪1), indicating no significant non-carcinogenic risk for adults. Metal contribution to risk: Zn > Pb > Ni > Mn > Hg > Fe > Cr > Cd > As (via ingestion) and Zn > Cd > As > Mn > Cr > Ni > Hg > Pb > Fe (via dermal exposure). On the other hand, for children, all metals showed HQ < 1 and the Hazard Quotient for the ingestion and dermal (HI_total_) indicated a value of 9.22$\:\times\:$10^−4^ (≪1) (Fig. 6B), indicating no significant non-carcinogenic risk for adults. Metal contribution to risk: Zn > Pb > Ni > Mn > Hg > Fe > Cr > Cd > As (via ingestion) and Zn > Cd > As > Mn > Cr > Ni > Hg > Pb > Fe (via dermal exposure).

### Carcinogenic risks (HLCR)

#### Incremental lifetime cancer risk (HLCR)

**Fig. 7:**
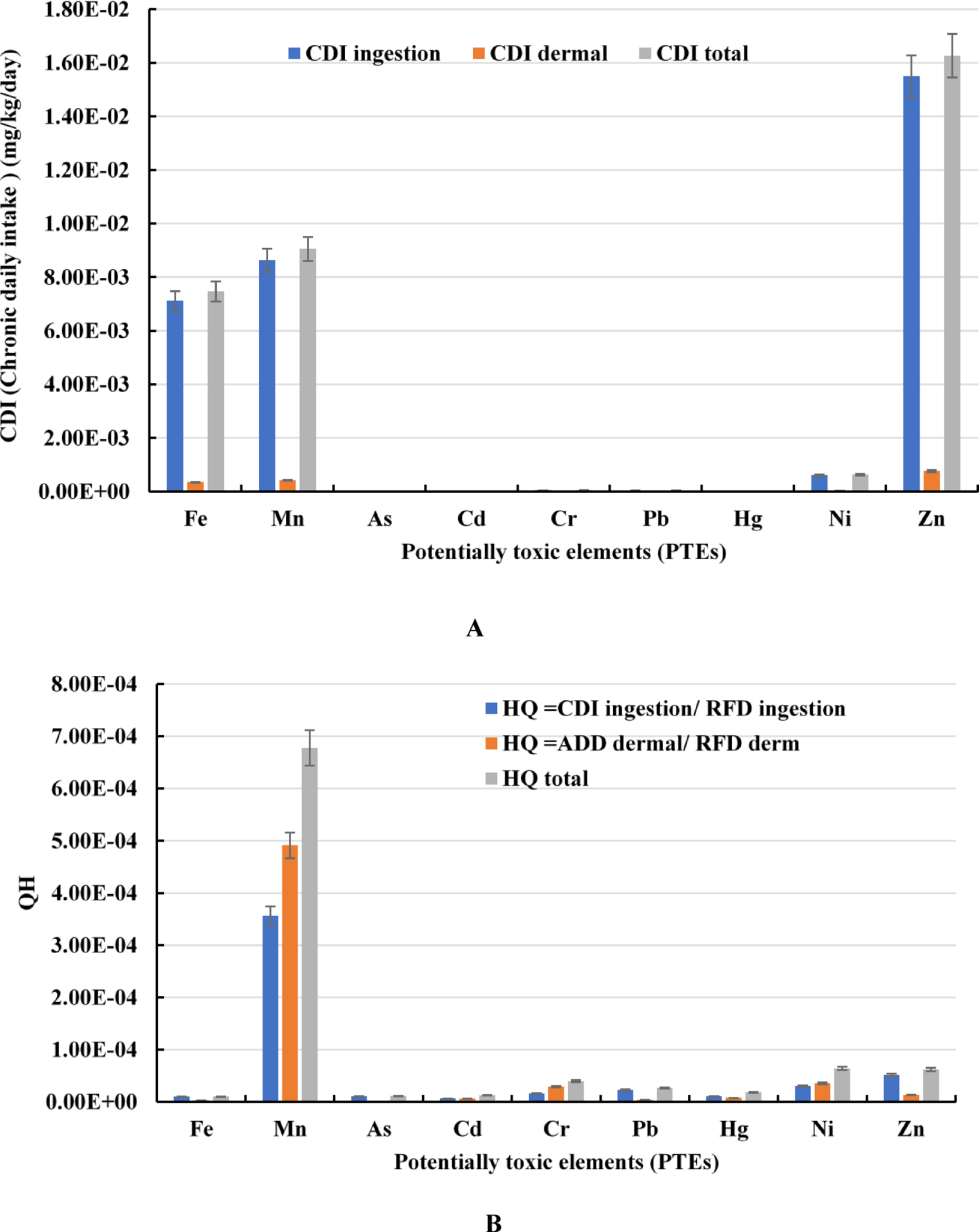
(**A**): Chronic daily intake (CDI) and (**B**): hazard quotient (HQ) via ingestion and dermal for children.

For adults, the Ni posed the highest risk (avg: 1.23 $\:\times\:$ 10⁻^3^), exceeding the USEPA threshold (1 $\:\times\:$ 10⁻^4^). Cd showed the lowest risk (avg: 4.14 $\:\times\:$ 10⁻^6^). Cumulative exposure via oral/dermal pathways posed no significant cancer risk, though Cr, Cd, Pb, and Ni remain priority carcinogens^21,32^. On the other hand, for children, the Ni posed the highest risk (avg: 1.09 $\:\times\:$ 10⁻^3^), exceeding the USEPA threshold (1 $\:\times\:$ 10⁻^4^). Cd showed the lowest risk (avg: 1.52 $\:\times\:$ 10⁻^5^) (Fig. 7).

## Discussion

### Hydrochemical characteristics, spatial distribution, and sources of major ions in groundwater

The spatial distribution of major ions (HCO₃⁻, Cl⁻, SO₄²⁻, and NO₃⁻) in the study area reflects a complex interaction between natural hydrogeochemical processes and anthropogenic activities. Bicarbonate (HCO₃⁻) shows relatively high and consistent concentrations across the study area, indicating its dominance in groundwater chemistry and suggesting widespread carbonate weathering as a primary geochemical control. In contrast, chloride (Cl⁻) and sulfate (SO₄²⁻) exhibit noticeable spatial variability, with relatively elevated concentrations observed in zones influenced by human activities. These variations highlight localized contamination sources and differences in recharge and flow conditions within the aquifer. Nitrate (NO₃⁻) concentrations, although generally low, show distinct spatial fluctuations, suggesting point-source inputs rather than uniform distribution. The geochemical behavior of these ions is governed by dissolution, ion exchange, and transport processes within the aquifer system. Carbonate dissolution contributes significantly to HCO₃⁻ levels, while sulfate is partly derived from the dissolution of evaporitic minerals such as gypsum. Chloride behaves conservatively in groundwater and is commonly used as an indicator of contamination sources. Nitrate, being highly mobile and soluble, is particularly sensitive to surface-derived inputs and rapidly transported through the vadose zone into groundwater. These processes collectively define the hydrochemical facies and influence groundwater suitability for different uses. From an environmental perspective, the observed ion composition can be attributed to three main sources. First, agricultural activities play a significant role, where excessive application of fertilizers leads to nitrate enrichment, and irrigation return flow contributes to the accumulation of dissolved salts such as chloride and sulfate. Second, wastewater infiltration, including leakage from septic systems and unregulated disposal of domestic effluents, introduces additional loads of chloride and nitrate into the aquifer. Third, natural geochemical processes, particularly the dissolution of carbonate and evaporite minerals, are responsible for the baseline concentrations of bicarbonate and sulfate in groundwater. These findings are consistent with regional hydrogeochemical studies and emphasize the combined impact of anthropogenic pressures and natural controls on groundwater quality.

### A comparison between the current Qalyubia groundwater study and similar global research, synthesized from the search results

Water Quality Indices (WQI): The current study’s WQI range (22–36, “Good”) aligns with assessments in Kerala, India (Satish Kumar et al. 2016), where 70% of samples were suitable for drinking. However, it contrasts sharply with Linfen Basin, China, where 33% of samples exceeded safety limits due to Pb, F⁻, and SO₄²⁻ pollution^38,39^. Heavy Metal Indices: Qalyubia’s low Heavy Metal Pollution Index (HPI ≤ 15) resembles “minimal pollution” in Telangana, India. Conversely, its Metal Index (MI: 0.81–3.29, “moderately affected”) mirrors Coimbatore, India, where industrial zones showed MI > 2.5 due to geogenic/anthropogenic fluoride^23,39^.

### Contamination profiles and sources

Anthropogenic vs. Geogenic Dominance: Qalyubia’s marginal Fe/Mn exceedances stem from natural aquifer leaching, similar to fluoride in Shanxi, China^38^. In contrast, studies near industrial belts (e.g., Coimbatore, India) attributed 60% of nitrate/fluoride pollution to textiles and smelting^23^. Microbiological Threats: Unlike Qalyubia (which focused on chemical parameters), Egyptian drains like the Bahr El-Baqar exhibited severe microbial contamination (fecal coliforms in 43% of wells), highlighting regional neglect of sewage management^40,41^. The significantly higher QH value for Manganese (Mn) compared to other PTEs, as shown in Figs. 6B and 7B, indicates it is a primary pollutant of concern in the study area. This is likely attributed to the geogenic conditions of the alluvial aquifer in the Nile Delta, which are naturally anoxic and favor the reduction of Mn-oxides, leading to the mobilization of Mn²⁺ into the groundwater. Furthermore, anthropogenic activities such as the infiltration of irrigation return flows containing organic matter can enhance these reductive dissolution processes, exacerbating Mn concentrations.

### Health risk assessment variations

Non-Carcinogenic Risks: The absence of significant risks in Qalyubia diverges from Linfen Basin, where 80% of children faced fluoride/nitrate hazards^39^. Children’s heightened susceptibility (2–3× higher than adults) was consistent globally, driven by greater water intake/kg body weight and developmental vulnerabilities^42,43^. Carcinogenic Threats: Qalyubia reported no carcinogenic concerns, whereas Linfen Basin identified Cr⁶⁺ and Cd as key carcinogens, with 100% of samples posing risks^38,39^.

### Irrigation suitability challenges

Salinity vs. Sodicity: Qalyubia’s irrigation indices indicated “good” quality (low Na⁺), akin to Santuri, India. However, the Bahr El-Baqar drain (Egypt) fell into “high salinity” due to agricultural runoff, restricting use in poorly drained soils^41,43^. Heavy Metal Transfer: Unlike Xinzhou, China, where Cd accumulation in crops exceeded safety limits, Qalyubia did not evaluate soil-crop transfer as a critical gap for agricultural planning^39^.

### Climate variability and its effects on groundwater quality and recharge

Climate variability plays a significant role in controlling groundwater recharge and quality, particularly in arid and semi-arid regions such as Egypt. Variations in precipitation patterns, coupled with increasing temperatures, directly influence recharge rates to aquifer systems. Reduced rainfall and higher evapotranspiration lead to limited natural recharge, which in turn increases the concentration of dissolved ions in groundwater. This process enhances salinization and deteriorates water quality, especially in shallow alluvial aquifers of the Nile Delta^44–46^. Furthermore, prolonged dry periods reduce dilution capacity, allowing contaminants such as nitrates and chlorides to accumulate. Similar findings were reported by Abd-Elaty et al.^47^, who highlighted that climate-induced stress on groundwater systems in Egypt contributes to declining water quality and increased vulnerability of aquifers to pollution. In addition to recharge reduction, climate variability also alters groundwater flow dynamics and geochemical processes within the aquifer. Rising temperatures and changing hydraulic gradients can accelerate mineral dissolution and ion exchange reactions, thereby modifying groundwater chemistry over time. In coastal areas, sea level rise associated with climate change further exacerbates groundwater salinization through seawater intrusion, increasing concentrations of chloride and sulfate. Eltarabily et al.^48^ emphasized that the combined effects of climate variability and anthropogenic activities, such as over-abstraction and agricultural practices, significantly impact groundwater sustainability in the Nile Delta region. These findings underscore the importance of integrating climate considerations into groundwater management strategies, including continuous monitoring, adaptive recharge enhancement, and protection of vulnerable aquifer zones.

### Socioeconomic and policy implications

Monitoring Gaps: Similar to rural Arizona wells^42^, Qalyubia’s reliance on homeowner testing risks under-detection of contaminants like Mn. India’s COVID lockdown underscored how industrial regulation improves water quality (33% nitrate decline)^23^, reinforcing Qalyubia’s call for stricter oversight. Remediation Strategies: Qalyubia’s “immediate remediation” aligns with WHO’s push for water safety plans. However, Egypt’s El-Qalyubia drains show policy fragmentation, as agricultural reuse continues despite known pollutants^41,49^.

### Regional vulnerability and climate interactions

 Qalyubia’s location within the Nile Delta provides relativily favorable water availability compared with the semiarid regions, reducing the severity of groundwater scarcity pressures.For example, in Xinzhou (China), approximatly 70% dependence on groundwater has been reported to increase vulnerability to pollution expousure and water quality deterioration ^39^. In contrast, groundwater in Qalyubia benefits from continuous recharge associated with the Nile Delta irrigation system, which helps maintain generally good water quality, as refelected by the WQI (Fig. 5) and irrigation indices (Table 7). Nevertheless, the moderate Metal Index (MI) values observed in this study indecate the presence of potentiallty toxic elements that require attention. Climate variability, including rising temperatures and increased evapotranspiration, may futher concentrate dissolved constituents and trace metals through reduced diluation and recharge. Therefore, continuous monitoring and adaptive groundwater manegement are essential to prevent future deterioration of groundwater quality and protect public health.

### Synthesis and research gaps

The Qalyubia study reflects a “moderately affected” groundwater system with lower risks than industrial zones but higher vulnerability than pristine aquifers. Critically, it shares limitations with similar Egyptian studies (e.g., unassessed microbial/mixture toxicity^40^. Future work must: (i) expand monitoring: Include emerging contaminants (e.g., pesticides) and seasonal dynamics, as demonstrated in monsoon-sensitive regions^23,49^, (ii) Cross-media transfer: Evaluate groundwater-soil-crop pathways, modeled after Xinzhou’s integrated assessments^39^, and (iii) Policy integration: Adopt WHO’s water safety plans, leveraging Coimbatore’s success in pollution reduction via industrial controls^23,49^. This comparison underscores that while Qalyubia’s groundwater is currently secure, its moderate metal pollution and Egypt’s rapid urbanization necessitate preemptive management, a lesson from global counterparts where delayed action escalated risks.

### Final outcomes of the study and how it will reach society?

Validation of current suitability: Groundwater in Qalyubia is currently suitable for drinking (WQI = 22–36, “Good”) and irrigation based on tested parameters and non-carcinogenic risk assessment.

Identification of emerging threat: The Metal Index (MI = 0.81–3.29) provides a clear warning of moderate cumulative metal pollution (Fe, Mn, Cd, As, Cr, Pb, Hg, Ni, Zn), even though individual metals (except minor Fe/Mn exceedances) and HPI suggest low immediate risk.

Vulnerability assessment: Confirmation that children face higher susceptibility to potential contaminants than adults.

Call to action: The study concludes that proactive monitoring and remediation are urgently needed to prevent further deterioration and protect public health and agricultural sustainability.

How these outcomes reach society & create impact:

Informing policy & regulation: government agencies: Findings are presented to the Egyptian Ministry of Water Resources and Irrigation, the Ministry of Health, and the Environmental Affairs Agency. This provides scientific evidence to:

Strengthen monitoring programs: Mandate regular, targeted testing of PTEs in Qalyubia’s groundwater wells, especially where MI was highest.

Develop remediation plans: Allocate resources for treating contaminated wells (e.g., filtration for Fe/Mn) or identifying alternative safe water sources. The remediation of contaminated groundwater in the study area requires the implementation of integrated and sustainable approaches that address both point and diffuse pollution sources. In-situ remediation techniques have proven to be particularly effective under hydrogeological conditions similar to those of the Nile Delta. Among these, permeable reactive barriers (PRBs) represent a promising solution, as they enable passive treatment of groundwater through natural flow processes. PRBs filled with reactive materials such as zero-valent iron, activated carbon, or natural media can effectively remove nitrates, iron, and manganese through mechanisms including adsorption, reduction, and precipitation. Recent work by Meky et al.^50–52^ demonstrated that PRBs are cost-effective and environmentally sustainable systems suitable for large-scale application in alluvial aquifers. In addition, reactive well systems, as highlighted by Meky^50,51^, can enhance in-situ remediation by promoting groundwater circulation and facilitating contaminant degradation through geochemical reactions. These systems are particularly useful in areas with localized contamination and limited accessibility. In addition to in-situ methods, ex-situ and preventive measures are essential for ensuring safe water supply and long-term groundwater protection. Point-of-use treatment systems, such as aeration and filtration units, can effectively remove iron and manganese from contaminated wells, making groundwater suitable for domestic use. Furthermore, managed aquifer recharge (MAR) techniques can improve groundwater quality by diluting contaminant concentrations and enhancing natural attenuation processes, provided that recharge water is adequately treated. Controlling pollution at the source remains a key priority; this includes optimizing fertilizer application, adopting precision agriculture practices, and improving wastewater management to reduce nitrate and salinity inputs into the aquifer. The identification of alternative safe water sources, along with the implementation of continuous monitoring systems supported by GIS tools, is also crucial for sustainable groundwater management. These combined approaches align with recent research emphasizing the importance of integrating remediation technologies with proactive management strategies to mitigate groundwater contamination under increasing environmental and climatic pressures.

Update standards/guidelines: Inform potential revisions to national water quality standards based on cumulative risk (MI concept).

Local authorities (Qalyubia Governorate): Enable targeted local action plans and budget allocation for well maintenance and pollution source control.

Guiding water management & infrastructure:

Water utilities/providers: Results guide decisions on well selection, water blending strategies, and potential infrastructure upgrades (e.g., installing specific treatment technologies in affected areas).

Agricultural cooperatives: Irrigation suitability confirmation supports sustainable farming, while the metal warning prompts awareness about potential long-term soil accumulation risks.

Raising public awareness & protection:

Public health campaigns: Health authorities and NGOs use the findings to:

Reassure communities about current safety (mitigating unwarranted fear).

Highlight the specific risk to children, promoting protective measures (e.g., continued use of treated water for infants where available, awareness of symptoms related to metal exposure).

Educate the public on the early warning nature of the moderate pollution finding and the importance of remediation efforts.

Community engagement: Local leaders and NGOs disseminate simplified findings, explaining why monitoring and potential future actions (like well treatment) are necessary for long-term safety.

Scientific foundation for future action:

Source identification: The study prompts further research to identify why metals are accumulating (e.g., industrial discharge, geological leaching, sewage infiltration).

Long-term tracking: Establishes a baseline for future studies to track pollution trends and measure the effectiveness of interventions.

Broader application: The methodology (especially using WQI, MI, and HPI together) serves as a model for assessing groundwater in similar urban, densely populated delta regions globally.

In essence, the study translates scientific data into actionable intelligence: It provides reassurance about current use, sounds an early alarm about a growing problem (cumulative metal pollution), identifies the most vulnerable group (children), and provides the scientific justification for authorities and communities to invest in monitoring and remediation before the water becomes unsafe. This directly contributes to protecting public health, ensuring sustainable agriculture, and safeguarding a critical water resource for a heavily populated region.

## Conclusion

This study provides a comprehensive assessment of groundwater quality, suitability, and health risks in Qalyubia Governorate, a highly populated region of Egypt’s Nile Delta. Groundwater is currently suitable for drinking and irrigation, as indicated by good Water Quality Index values (23–38) and favorable irrigation indices, with sodium (Na⁺) dominating the hydrochemical composition. However, the Metal Index indicates a moderately affected status due to localized iron and manganese enrichment, signaling emerging vulnerability to anthropogenic pressures. Health risk assessment confirms no significant carcinogenic or non-carcinogenic risks under present conditions, although children represent the most sensitive population group. These results establish a robust geochemical and health-risk baseline and provide an early warning for groundwater degradation. From a policy perspective, the study recommends: (i) targeted remediation of Fe and Mn hotspots, (ii) implementation of continuous groundwater quality monitoring focused on trace metals, and (iii) integration of geochemical baselines and health-risk tools into regional water safety plans under an Integrated Water Resources Management (IWRM) framework. These measures are essential to ensuring long-term groundwater security in the Nile Delta.

## Data Availability

The data are available from the corresponding author upon reasonable request.
